# Evaluation of Intravenous Administration of Anti-Infective Agents and Documentation Quality in Orthopedics and Trauma Surgery: A Quantitative Study on Discrepancies Between Physician Prescriptions and Nursing Records

**DOI:** 10.3390/antibiotics15040341

**Published:** 2026-03-27

**Authors:** Anna-Judith Dahse, Laura Klimpel, Katrin Heinitz, Martina P. Neininger, Christoph Lübbert, Annett Huke, Christian Kleber, Andreas Roth, Christina Pempe, Dmitry Notov, Pierre Hepp, Christoph-Eckhard Heyde, Thilo Bertsche, Yvonne Remane

**Affiliations:** 1 Hospital Pharmacy, Leipzig University Medical Center, 04103 Leipzig, Germanyyvonne.remane@medizin.uni-leipzig.de (Y.R.); 2 Drug Safety Center, Medical Faculty, Leipzig University and Leipzig University Medical Center, 04103 Leipzig, Germany; 3 Department of Clinical Pharmacy, Institute of Pharmacy, Medical Faculty, Leipzig University, 04103 Leipzig, Germany; 4 Department of Neuropediatrics, Center for Children and Adolescents, University Medicine Greifswald, 17489 Greifswald, Germany; 5 Division of Infectious Diseases and Tropical Medicine, Department of Medicine I, Leipzig University Medical Center, 04103 Leipzig, Germany; 6 Interdisciplinary Center for Infectious Diseases, Leipzig University Medical Center, 04103 Leipzig, Germany; 7 Department of Orthopedic, Trauma and Plastic Surgery, Leipzig University Medical Center, 04103 Leipzig, Germany

**Keywords:** antibiotics, anti-infectives, administration routes, intravenous, application frequency, documentation, medical records

## Abstract

**Background:** Intravenous anti-infectives are an important part of postoperative care, but discrepancies between prescribed and documented administrations remain widespread and require systematic evaluation. **Methods:** In an exploratory study, prescribed and documented intravenous anti-infective administrations were retrospectively analyzed using patient charts, digital nursing reports and, in cases of deviations, consultations with the responsible staff. The discrepancies were classified into three categories: (I) documentation, (II) administration, and (III) a combination of both. The relationship between discrepancies and dosing interval, time of administration (weekday and shift assignment), and intravenous administration route was statistically analyzed (Χ^2^ test, residual analysis). **Results:** Of 5016 anti-infective administrations in 219 patients, 1135 (22.6%) had at least one discrepancy, of which 68.2% (774 of 1135) belonged to category I. Significant differences in the frequency of discrepancies between surgical wards and the dosing intervals were observed. On weekdays, 23.6% of drug administrations (832 of 3519) showed discrepancies compared to 20.2% on weekends (303 of 1497, OR = 1.22, 95% CI 1.05–1.42, *p* = 0.008). Although the early shift had the lowest administration rate, it showed significantly more discrepancies than expected (313.6 expected vs. 553 observed; adjusted standardized residual +18.1; *p* < 0.001). Drug administration via the peripheral venous route was more susceptible to discrepancies than the central venous administration route (23.2% [963 of 4149] vs. 19.8% [172 of 867]), OR 1.18; 95% CI 1.01–1.38; *p* = 0.031). **Conclusions:** Approximately a quarter of anti-infective administrations were affected by discrepancies, predominantly in category I, with the highest incidences occurring during the early shift and on weekdays. This requires a multi-step improvement program.

## 1. Introduction

Intravenously administered anti-infectives represent a core element of surgical care, both preventively and therapeutically [1,2]. In particular, serious infections of the musculoskeletal system, such as periprosthetic joint infections or vertebral osteomyelitis, are associated with a significantly increased mortality rate [3,4]. To treat these infections, anti-infective therapy is typically started intravenously to achieve therapeutically relevant plasma levels rapidly [5,6]. Due to this clinical relevance, complete and timely documentation of intravenous anti-infective drug administration is crucial, as precise administration times influence immediate therapeutic dosing decisions [7]. In addition to the selection of drugs and dosage, the effectiveness of the treatment is significantly influenced by its correct dosage interval, as the efficacy of many drugs is significantly influenced by their pharmacokinetics and pharmacodynamics. This is particularly evident for drugs whose therapeutic effectiveness is time-dependent, such as β-lactams [8].

According to the “German Guideline on Strategies to Ensure Rational Antibiotic Use in Hospitals” [9], reliable and structured documentation is an essential prerequisite for safe antimicrobial therapy, as it enables transparent clinical decision-making, supports interdisciplinary communication, and forms the basis for continuous evaluation of indication, dosing, treatment duration, de-escalation strategies and therapeutic outcomes. This approach renders the objectives of antibiotic stewardship (ABS) comprehensible and amenable to daily management in the ward environment. Numerous hospitals have established ABS programs to ensure the proper use of anti-infectives and to guarantee the quality of documentation [9,10,11,12]. Despite the established ABS structures, the correct administration of intravenous drug therapy poses a considerable challenge due to the condensed workflows on the ward, which increase the risk of administrative and documentation discrepancies [13,14]. Premature signatures or subsequent collective documentation increase this risk [15].

The aim of this study was therefore to investigate the discrepancies between physician prescriptions and nursing documentation of intravenous anti-infective drug administration in surgical wards of a University Hospital, categorizing them into (I) documentation discrepancies, (II) drug administration discrepancies, and (III) a combination of both (documentation and administration). We hypothesized that discrepancies between the prescribed and documented administration of anti-infective agents are related to aspects of the workflow, focusing in particular on procedural factors such as nursing shift, day of the week, and route of administration.

## 2. Results

### 2.1. Patient Characteristics and Administered Anti-Infectives

**Figure 1:**
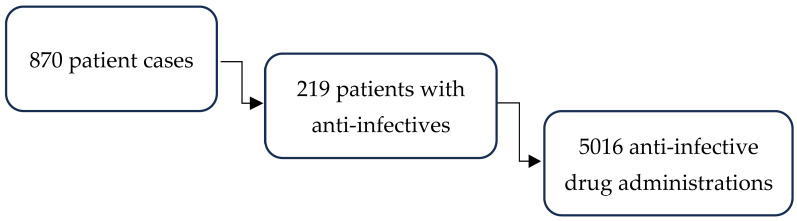
Flow diagram of the study population.

During the study period, a total of 870 consecutive patient cases were registered, of which 219 patients (25.2%) with a total of 5016 anti-infective drug administrations were included in the study (Figure 1). No patients were excluded.

The study was conducted across four units, each specializing in different surgical disciplines: trauma surgery, spinal surgery, septic surgery, and orthopedics, including arthroscopic and specialized joint surgery as well as sports injuries. Baseline characteristics are shown in Table 1.

Although the units differed in terms of their surgical specialties, their patients, and their use of anti-infectives, they were treated as a single cohort in the following analysis, as the focus was on identifying the causes and processes underlying the discrepancies between physicians’ prescriptions and nursing staff records.

The patient cohort comprised 114 females (52.1%) and 105 males (47.9%). The median age of the patients was 68 years (Q25/Q75: 57/83). The most frequently administered anti-infective drugs were cefazolin (*n* = 984 doses; 19.6%), a first-generation cephalosporin with good efficacy against Staphylococcus aureus and other Gram-positive pathogens, and ampicillin/sulbactam (*n* = 978 doses; 19.5%). Cefotaxime (*n* = 748 doses; 14.9%), benzylpenicillin (*n* = 415 doses; 8.3%) and piperacillin/tazobactam (*n* = 332 doses; 6.6%) were also widely used. Meropenem was administered with lower frequency (*n* = 276 doses; 5.5%). A comprehensive overview of the prescribed anti-infective agents is given in Table 2.

### 2.2. Discrepancies in the Administration of Anti-Infective Therapy

A total of 5016 intravenous anti-infective drug administrations were evaluated, of which 3881 (77.4%) were documented and administered correctly. One discrepancy was identified in 1096 of 5016 drug administrations (21.9%), two discrepancies in 38 of 5016 administrations (0.76%), and three discrepancies in one of 5016 administrations (0.02%). Thus, a total of 1135 anti-infective administrations had discrepancies, which corresponds to 22.6% of the 5016 anti-infectives administered. Of these 1135 discrepancies, 68.2% (774 of 1135) belonged to category I (documentation). A further 24.2% (275 of 1135) were related to category II (administration), while 2.2% (25 of 1135) were classified as category III (both documentation and administration). In 5.4% of administrations (61 of 1135), it was not possible to determine a clear assignment retrospectively.

#### 2.2.1. Unit Relationship

The analysis revealed that, following Bonferroni correction, significant differences in discrepancy rates between the units were observed, with lower error rates in Unit A and higher error rates in Units C and D. Unit B did not differ significantly from the expected value after adjustment for multiple testing (Table 3).

#### 2.2.2. Dosing Interval Relationship

The majority of anti-infectives (44.9%; 2251 of 5016) were administered at 8 h dosing intervals, followed by 4 h dosing intervals (22.0%; 1104 of 5016) and 6 h dosing intervals (20.9%; 1050 of 5016). Anti-infective prescriptions with a 12 h dosing interval (6.9%; 347 of 5016) and a 24 h dosing interval (5.2%; 261 of 5016) were less common. Three doses could not be assigned to any interval: one was accidentally administered additionally, and two could not be assigned due to documentation discrepancies. We found significant differences in the frequency of discrepancies between the dosing intervals (Table 4).

In the 4, 6, and 8 h dosing intervals, category I discrepancies were predominantly identified: 70.9% (202 of 285), 62.8% (115 of 183), and 74.7% (424 of 568) of the affected drug administrations, respectively. In the 12 and 24 h intervals, category II discrepancies were detected in 52.0% (26 of 50) and 63.0% (29 of 46) of the affected drug administrations, respectively. Category III discrepancies were found to be less than 10% per dosing interval.

#### 2.2.3. Administration Time Relationship

##### Weekday

Of a total of 5016 drug administrations, 3519 (70.2%) were recorded from Monday to Friday (weekdays), and 1497 (29.8%) on the weekend. On average, around 90 anti-infectives were administered per weekday (Monday to Friday), compared to around 107 on each weekend day during the observed period. On weekdays, 23.6% (832 of 3519) of the administrations showed at least one discrepancy, compared to 20.2% (303 of 1497) on the weekend (OR = 1.22; 95% CI 1.05–1.42, *p* = 0.008). During the week, category I discrepancies were found in 65.1% (542 of 832) of the administrations, and category II discrepancies in 26.0% (216 of 832). On weekends, category I discrepancies were found in 76.6% (232 of 303) of the affected administrations, and category II discrepancies in 19.5% (59 of 303).

##### Nursing Shift

**Figure 2:**
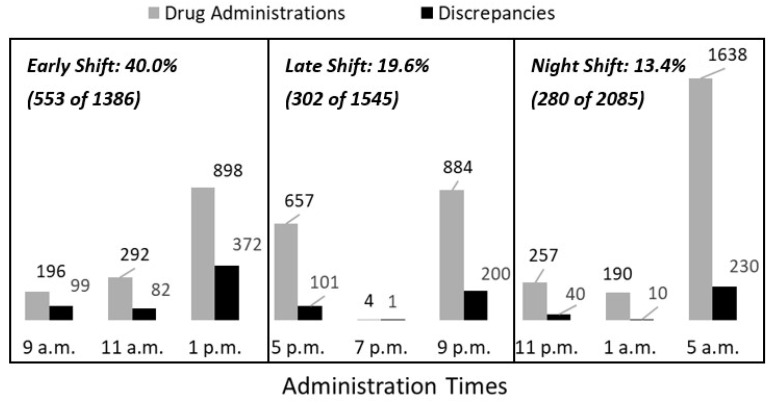
Distribution of drug administration times, drug administrations and discrepancies throughout the day and shift (*n* = 5016).

The analysis of administration times, administrations and corresponding discrepancies throughout the day revealed a marked tendency for temporal clustering, with the majority of administrations occurring during the early morning, early afternoon and late evening periods (Figure 2).

The majority of anti-infective drug administrations were made during the night shift (2085 of 5016; 41.6%). Nevertheless, the share of administrations with discrepancies was the highest during early shifts (553 of 1386; 40.0%). During the late and night shifts, 19.6% (302 of 1545) and 13.4% (280 of 2085) of administrations with discrepancies were recorded. Although the early shift had the lowest administration rate, it showed significantly more discrepancies than expected (313.6 expected vs. 553 observed; adjusted standardized residual +18.1; *p* < 0.001). In the late shift, 302 discrepancies of 1545 administrations were recorded (expected number: 349.6; adjusted standardized residual +3.5; *p* < 0.001), and in the night shift, 280 discrepancies of 2085 administrations were documented (expected number 471.8; adjusted standardized residual −13.1; *p* <0.001). During the early shift, category I discrepancies occurred in 433 of 553 affected administrations (78.3%), whereas category II discrepancies increased across shifts and were highest during the night shift (18.8% [104 of 553] vs. 20.2.% [61 of 302] vs. 39.3% [110 of 280]).

#### 2.2.4. Intravenous Administration Relationship (Peripheral vs. Central)

A total of 4149 out of 5016 analyzed anti-infective administrations (82.7%) were to be administered via the peripheral intravenous route, and 867 of 5016 (17.3%) via the central intravenous route. With regard to the peripheral route, 3186 of 4149 administrations (76.8%) were not affected by discrepancies, while 963 of 4149 (23.2%) showed discrepancies. A total of 695 of 867 administrations via central venous catheters (80.2%) were in accordance with the prescription, while 172 of 867 (19.8%) demonstrated at least one discrepancy. Administrations via the peripheral venous route were more susceptible to discrepancies than via the central administration route (23.2% (963 of 4149) vs. 19.8% (172 of 867), OR 1.18; 95% CI 1.01–1.38; *p* = 0.031).

When comparing the administration of anti-infectives via central vs. peripheral venous access, the majority of discrepancies were observed in category I (73.8% (127 of 172) and 67.2% (647 of 963), respectively). Category II discrepancies occurred in 25.8% (248 of 963) of drug administrations via peripheral venous access, and in 15.7% (27 of 172) of drug administrations via central venous access. In the nursing documentation, the lack of a peripheral venous access at the intended time of drug administration, the absence of the patient on the ward, or the patient’s refusal of anti-infectives were reported as main factors for administration-related discrepancies in cases of peripheral intravenous therapy. With regard to central venous administrations of anti-infectives, the absence of the patient was also reported as a reason, while explicit refusal of intravenous therapy was noted less frequently. Category III discrepancies were rare, with an incidence of 0.6% (1 of 172) in central venous administrations vs. 2.5% (24 of 963) in peripheral venous administrations. Overall, 61 administrations were not evaluable, comprising 17 of 172 via central venous access, and 44 of 963 via peripheral venous access.

## 3. Discussion

In this retrospective analysis of 5016 anti-infective drug administrations in 219 patients, at least one discrepancy was recorded in 22.6% (1135 of 5016); 68.2% (774 of 1135) of discrepancies were related to documentation discrepancies. Statistical analysis found significant differences between different surgical units, nursing shifts, weekdays and weekends, dosing intervals and types of venous access routes. Our results indicate that intravenous drug administration in general is associated with a high risk of discrepancies, depending on the medication process organization. Furthermore, the significant differences in discrepancy rates across the units suggest a link to variations in work organization. Given the exploratory nature of this analysis, the medication process in each department should be thoroughly examined and evaluated in future research.

Systematic reviews consistently report considerable discrepancies, particularly with regard to the appropriate drug administration time, dosage, and omission errors. The reported rates in the literature vary between 10% and 70%, depending on the study design and discrepancy definitions [16,17]. Therefore, the recorded discrepancy rate of 22.6% (1135 of 5016) in this study confirms previously published data.

According to Jheeta et al. [18], categorizing drug administration and documentation discrepancies provides a structured and comprehensible overview as well as a solid basis for further analysis and targeted investigations. In general, drug selection is influenced by various factors. These include international and local treatment guidelines, the in-house antimicrobial resistance situation, the suitability of the drug for each individual patient based on their medical history, and the pharmacological properties of the drug, such as kill kinetics, tissue penetration, potential collateral damage and side-effect profiles. In this study, cefazolin and ampicillin/sulbactam were the most commonly prescribed drugs, which reflects adherence to the hospital’s internal anti-infective guidelines (6th edition, October 2024 [19]), with its high-dose regimen (short dosing intervals) especially for bone and joint infections. Conversely, the few prescriptions for cefuroxime are consistent with the current in-house microbiological resistance situation.

The evaluation of dosing intervals showed the most discrepancies for the 4 and 8 h dosing intervals. The intervals largely coincide with periods of high work density, such as shift handovers, and align with results of earlier studies indicating that interruptions are a significant factor in drug administration errors [20]. Consequently, the observed discrepancy rate is significantly higher than expected during the early shift. During this period, scheduled surgeries, ward rounds, consultative examinations, diagnostic procedures, physiotherapy, and nursing activities occur concurrently, resulting in a high degree of process density. In contrast, the number of simultaneous tasks decreases during the night shift, while there are fewer staff members. Although intravenous administration is generally associated with higher discrepancy rates than oral administration, our data suggest that an increase in administration frequency (e.g., from 8 to 4 h dosing intervals) does not necessarily result in a higher discrepancy rate. This finding is consistent with the literature, which indicates that drug safety is primarily negatively affected by process density and not solely by dosing frequency [14,21,22].

Overall, category I discrepancies occurred most frequently in the early shift. This shift is associated with the highest staff workload, promoting these discrepancy rates. Nursing processes, including the preparation and administration of medications, were often concurrent with medical processes such as rounds and consultations [14]. As a result, doses were frequently authorized prematurely, retrospectively, or were inadvertently not administered [14,23]. The study identified a number of reasons, including prescriptions that were ambiguous (e.g., incomplete drug names, dosages, strengths, and past prescriptions), failure to complete mandatory fields in the paper chart, illegible handwriting, and the absence of a physician’s signature, e.g., in the case of verbal orders. As a direct consequence of this analysis and to address these procedural challenges, we plan to implement a multi-step improvement program: (1) Staff scheduling with two nurses jointly responsible for medication rounds, to improve adherence to drug administrations. (2) A mandatory four-eyes principle for all drug administrations, including verbal confirmation of the patient’s name and the prescribed drug, showing the drug and the signature approval promptly after administration. (3) Installing whiteboards on patient room doors, visible from the hallway, displaying scheduled medications.

In contrast to weekdays, a reduction in drug administration discrepancies was observed on the weekend, probably due to a lower process density and a lower number of planned examinations, and thus possibly increased time for cross-checking of prescriptions and drug administration. Conversely, an increase in category I discrepancies was observed, coinciding with reduced staffing capacities. Statistical analysis indicates that discrepancy rates during the early shift were significantly higher than expected, both on weekdays and on weekends. International evidence is heterogeneous: some studies reported elevated medication error rates in the early shift [24,25,26], while others found higher rates at night [27,28], depending on the setting, patient population, discrepancy type, and data collection methods. Fewer discrepancies were observed during the late shift and night shift, but still with a clinically relevant prevalence of 14–20%. This observation provides moderate statistical evidence but limited practical relevance, suggesting that the day of the week alone does not have a decisive influence. Finally, the results demonstrate that peripheral venous catheters were associated with more discrepancies, especially category II discrepancies. These findings are supported by a small number of international studies [29,30]. However, these studies highlight different risk profiles, but do not include systematic analyses. The moderate differences observed in this study should be evaluated with caution in terms of their practical relevance.

Therefore, priority should be given to measures that ensure effective access. Delays in intravenous cannula placement were frequently observed, often due to the limited availability of medical staff being occupied with surgeries. Consequently, from 2026 onwards, cannula placement at our institution will be performed by qualified non-medical staff. Furthermore, based on current study results and in close cooperation with the ABS team, early oralization concepts should be established for drugs with high bioavailability to improve timely drug administration and reduce nursing workload [31,32,33,34,35,36]. Evidence indicates that intravenous drug administration requires, on average, 10–20 min of nursing time per administration, whereas oral administration typically takes less than 2 min [37,38].

Ultimately, the main reason for the frequent discrepancies in the documentation was the way the medication process was organized. Since there was no electronic documentation, paper records were the limiting factor. A promising strategy is the implementation of a comprehensive electronic health record system with integrated computerized physician order entry and nursing documentation. Such systems have shown a reduction in transcription errors, to improve medication traceability, and to support standardized workflows for parenteral drug administration [39,40]. The results of this study highlight the necessity for prompt electronic documentation with precise time stamps and standardized feedback to the medical team [16,17]. Immediate documentation at the bedside and the possibility of simultaneous interprofessional work in electronic patient records reduce discrepancies that previously occurred due to missing or delayed updates to paper documentation. The implementation of such a system is planned for 2026 and will provide an important opportunity to allow an evaluation of its impact on the frequency of documentation and drug administration discrepancies, particularly in the context of intravenous anti-infective therapy and varying administration frequencies.

### Strengths and Limitations

This analysis has a number of strengths and limitations: (1) The analysis is based on a large empirical dataset, enabling robust overall estimates as well as subgroup analyses. (2) Documentation quality was assessed using a systematic cross-check of paper-based charts and electronic nursing records, minimizing documentation-related artifacts. (3) A standardized data collection protocol in combination with a three-level classification system allowed measurement and interpretation of discrepancies within the processes on the ward. (4) The retrospective chart review was descriptive and based on the documented practices. We were unable to evaluate either the clinical appropriateness of drug administration, the outcomes, or the nursing work density. Shadowing and direct monitoring on the wards were not performed, even though these procedures are often considered a gold standard for identifying administration-related issues. As drug administration was not directly observed, the correspondence between chart documentation and real drug administration remains uncertain. Therefore, our conclusions are based on the available documentation, including plausibility assessments, cross-referencing, and checks for formal completeness. (5) The data collection slots for analysis were conducive to the recording of discrepancies during the early shift, which may have inflated the observed discrepancies in this shift, especially category I discrepancies (documentation). Nevertheless, the overall shift relationship remains interpretable. (6) In case of unexplained deviations, the responsible nursing staff was consulted in order to verify documentation-related artifacts and clarify any causes that carry the risk of recall bias. In addition, direct data collection on the wards may result in a Hawthorne effect, i.e., a temporary adjustment in the behavior of medical staff due to observation. (7) For the statistical analysis, drug administrations were treated as independent observations, although clustered within patients. We chose this approach as we were mainly interested in exploring the individual administration processes rather than determining effects on patient outcomes. As this was an exploratory study, statistical significance should be considered with caution. For this reason, we also refrained from multivariate analysis.

## 4. Materials and Methods

### 4.1. Study Design and Study Population

The retrospective analysis of routine data included consecutive adult patients (≥18 years) who received at least one prescribed intravenous anti-infective drug. Patients were assessed on the basis of the clinical documentation in their paper-based patient charts. The evaluation was performed between 2 January 2025 and 11 March 2025, aiming to evaluate a minimum of 4000 anti-infective administrations. Using the prior literature reporting discrepancy rates between 10% and 70%, we conservatively assumed an expected discrepancy rate of 25%. For a 95% confidence level and a desired precision of ±1.5%, the required sample size was approximately 3200 administrations. To allow for subgroup analyses, potential missing data, and sufficient expected cell counts for chi-square tests, the sample size was increased to ≥4000 administrations. The following aspects were analyzed to investigate the consistency between physician prescriptions and nursing documentation: (1) Do different dosing intervals influence the frequency of discrepancies? (2) Are the observed discrepancies related to the time of administration, taking into account both the shift and the day of the week? (3) Does the type of intravenous administration route (central venous catheter or peripheral venous access) influence the occurrence of discrepancies?

### 4.2. Study Setting

The Leipzig University Medical Center is a tertiary medical care facility in Saxony, Germany, with 1451 beds in 46 specialist departments (clinics, polyclinics, institutes, and departments) covering the entire medical spectrum. The study included four units of the Department of Orthopedic, Trauma and Plastic Surgery: trauma surgery, spinal surgery, septic surgery and orthopedics, including arthroscopic and specialized joint surgery/sports injuries. In these wards, the treatment of infections of the skin and soft tissues, bones and joints, as well as infections of the urinary and the respiratory tracts, and bloodstream infections were primarily managed by intravenous administration of anti-infectives. The study focused on the process of documentation as a routine procedure. The documentation was reviewed retrospectively using routinely recorded data from paper-based patient charts and electronic nursing reports. In accordance with the legal requirements of Section 29 of the Saxon State Hospital Act, written informed consent was not required. This approach was unable to detect administrations without a corresponding prescription, as the nursing staff was not shadowed during drug administration.

The patient chart was available in paper form, where all prescriptions were written in designated fields: drug name, dosage form, dose, and name of the medicinal product. The intravenously administered drug was prescribed in designated sections and had to be dated and signed by the responsible physician. Any changes, including dose adjustments, required re-entering the chart. The responsible nurse had to confirm the administration immediately with a handwritten signature in the patient’s chart, while the electronic nursing report was documented as continuous text in the hospital information system SAP IS-H med, version 8000.1.16.1161 (SAP SE, Walldorf, Germany). The work on the ward is organized in a three-shift system: early shift (6:30 a.m. to 2:30 p.m.), late shift (2:30 p.m. to 10:30 p.m.), and night shift (10:30 p.m. to 6:30 a.m.). The key content of the report included events and observations during the day and night shifts, special occurrences or changes in the patient’s condition. In addition, discrepancies in care and drug administrations were also recorded for transparency and traceability.

### 4.3. Data Collection and Evaluation

A systematic analysis was performed to compare physician prescriptions with nursing documentation. The corresponding data collection was done twice a day, in the morning and in the afternoon, by a designated pharmacist on the ward. This process recorded potential delays or omissions in nursing and medical documentation, including all patients. To ensure data quality, the retrospective evaluation was limited to previously scheduled administrations during the current shift and the two previous shifts, taking into account the practiced three-shift system. This methodological approach ensured that, despite the retrospective nature of the chart review process, the documentation was evaluated as promptly and as validly as possible.

The following data were recorded using Microsoft Excel LTSC for Windows, version 2408 (Microsoft Corporation, Redmond, WA, USA): ward, patient name, sex, age, case number, date and time of data collection, patient isolation status, prescribed drug, prescribed dosage, indications for therapeutic drug monitoring, type of venous administration route, date and time of administration, prescribed dosing schedule, and documented administration based on the signature of the nursing staff. Finally, the medication administered as documented in the patient’s chart was compared with the physician’s prescriptions. Discrepancies and their causes were subsequently cross-checked in the digital nursing report. In case of deviations, the responsible nursing staff was consulted in order to verify documentation-related artifacts and clarify any causes.

Discrepancies were classified according to a three-tier system: (1) category I includes documentation discrepancies with no direct impact on the actual administration. These comprise missing documentation despite administration of the anti-infective agent or premature documentation. (2) Category II covers administration discrepancies. These include delayed or omitted administrations (e.g., due to patient absence or refusal), extravascular administrations requiring subsequent restoration of venous access, or drugs that were not available at the scheduled time, as confirmed by nursing staff or reports. (3) Category III includes combined documentation and administration discrepancies, i.e., cases in which categories I and II occur simultaneously. For instance, a pre-signed record (documentation) was subsequently refused by the patient or ran paravascularly (administration).

### 4.4. Statistical Analysis

Descriptive statistics were compiled, and inferential statistical methods were used to identify significant relationships. Statistical analysis was conducted using SPSS for Windows, version 29 (IBM, Armonk, NY, USA). Depending on the normality of the distribution, metric variables are presented as median [25th/75th percentiles]. Two-sided Pearson’s chi-square tests were performed to analyze the differences in the occurrence of discrepancies between physicians’ prescriptions and nurses’ administration related to (i) administration time, (ii) dosing intervals, and (iii) intravenous route. A *p*-value < 0.05 was considered to indicate significance. For dichotomous variables, we calculated odds ratios (OR) and 95% confidence intervals (95% CI). For non-dichotomous nominal variables, post hoc tests were run. We calculated adjusted standardized residuals to identify statistical differences between observed and expected frequencies. Values exceeding ±1.96 are generally interpreted as indicating a significant deviation from expected counts for α = 0.05. Bonferroni corrections were calculated to adjust α for multiple testing.

### 4.5. Ethics Approval

The study was conducted in accordance with the ethical guidelines of the 1964 Declaration of Helsinki and its later amendments and was approved by the local ethics committee (Ethics Committee of the Medical Faculty, Leipzig University, Germany; registration number 430/24-ek, 30 January 2025).

## 5. Conclusions

In summary, this analysis reveals relevant discrepancies between physician prescriptions of anti-infectives and their documentation of administration by nursing staff. The discrepancies are primarily related to category I (documentation). They vary depending on the shift and the time of administration. The highest rate of discrepancies occurred during the week in the early shift, regardless of the day, coinciding with the peak of assumed work density. These findings suggest structural and process-related factors influencing everyday ward routines. Consequently, a multi-step improvement program should be considered and prospectively evaluated in clinical practice, comprising a mandatory four-eyes principle, the delegation of cannula placement to qualified non-medical staff, the establishment of concepts for early oralization, and the digitalization of the medication process, which is gaining strategic importance.

## Figures and Tables

**Table 1:** Baseline characteristics of surgical units.

Parameter	Unit A	Unit B	Unit C	Unit D
Number of patients	104	187	372	207
with anti-infective drug administration	81	45	42	51
with discrepancies in anti-infective drug administration	71	39	39	45
Number of anti-infective drug administrations	2421	830	498	1267
with discrepancies	477	165	162	331
Case mix index (CMI)	2.062	1.640	1.175	1.656
Average length of stay (days)	16.8	9.4	5.6	9.8

**Table 2:** Overview of prescribed intravenous anti-infectives (*n* = 5016).

Anti-Infective Agent	Drug Administrations (*n*, %)
Cefazolin	984 (19.6%)
Ampicillin/Sulbactam	978 (19.5%)
Cefotaxime	748 (14.9%)
Benzylpenicillin	415 (8.3%)
Piperacillin/Tazobactam	332 (6.6%)
Meropenem	276 (5.5%)
Clindamycin	238 (4.7%)
Flucloxacillin	197 (3.9%)
Vancomycin	166 (3.3%)
Ceftazidime	108 (2.2%)
Imipenem/Cilastatin	95 (1.9%)
Rifampicin	92 (1.8%)
Others	387 (7.7%)

**Table 3:** Observed and expected discrepancies by unit, adjusted standardized residuals (ASR), and *p*-values (*n* = 5016).

Unit	Frequency of Observed Discrepancies (%)	Expected Counts	ASR	*p* Value *
A	477/2421 (19.7%)	547.8	−4.8	<0.001
B	165/830 (19.9%)	187.8	−2.1	n.s. **
C	162/498 (32.5%)	112.7	+5.6	<0.001
D	331/1267 (26.1%)	286.7	+3.4	<0.001

* Bonferroni correction was applied, adjusted *p*-value to indicate significance: 0.005. ** not significant

**Table 4:** Observed and expected discrepancies by dosing interval, adjusted standardized residuals (ASR), and *p*-values (*n* = 5016).

Dosing Interval [h]	Frequency of Observed Discrepancies (%)	Expected Counts	ASR	*p* Value *
24	46/261 (17.6%)	58.9	−2.0	0.046
12	50/347 (14.4%)	78.4	−3.8	<0.001
8	568/2251 (25.2%)	508.3	+4.1	<0.001
6	183/1050 (17.4%)	237.1	−4.6	<0.001
4	285/1104 (25.8%)	249.3	+2.9	0.004
Not assignable	3/5016 (0.06%)	-	-	-

* Bonferroni correction was applied, adjusted *p*-value to indicate significance: 0.005.

## Data Availability

Anonymized data will be available upon request. Owing to the retrospective observational design of the study, the dataset originates from routine clinical records containing sensitive information and therefore cannot be fully shared publicly. Contact yvonne.remane@medizin.uni-leipzig.de.
